# Impact of a Complex Food Microbiota on Energy Metabolism in the Model Organism *Caenorhabditis elegans*

**DOI:** 10.1155/2015/621709

**Published:** 2015-04-19

**Authors:** Elena Zanni, Chiara Laudenzi, Emily Schifano, Claudio Palleschi, Giuditta Perozzi, Daniela Uccelletti, Chiara Devirgiliis

**Affiliations:** ^1^Department of Biology and Biotechnology “C. Darwin”, Sapienza University of Rome, Piazzale Aldo Moro 5, 00185 Rome, Italy; ^2^Food & Nutrition Research Center (CRA-NUT), Agricultural Research Council, Via Ardeatina 546, 00178 Rome, Italy

## Abstract

The nematode *Caenorhabditis elegans* is widely used as a model system for research on aging, development, and host-pathogen interactions. Little is currently known about the mechanisms underlying the effects exerted by foodborne microbes. We took advantage of *C. elegans* to evaluate the impact of foodborne microbiota on well characterized physiological features of the worms. Foodborne lactic acid bacteria (LAB) consortium was used to feed nematodes and its composition was evaluated by 16S rDNA analysis and strain typing before and after colonization of the nematode gut. *Lactobacillus delbrueckii, L. fermentum*, and *Leuconostoc lactis* were identified as the main species and shown to display different worm gut colonization capacities. LAB supplementation appeared to decrease nematode lifespan compared to the animals fed with the conventional *Escherichia coli* nutrient source or a probiotic bacterial strain. Reduced brood size was also observed in microbiota-fed nematodes. Moreover, massive accumulation of lipid droplets was revealed by BODIPY staining. Altered expression of *nhr-49, pept-1, and tub-1* genes, associated with obesity phenotypes, was demonstrated by RT-qPCR. Since several pathways are evolutionarily conserved in *C. elegans*, our results highlight the nematode as a valuable model system to investigate the effects of a complex microbial consortium on host energy metabolism.

## 1. Introduction

Fermented foods result from the metabolic activity of complex and heterogeneous bacterial communities, which proliferate within the food matrix using carbohydrate substrates to carry out fermentation processes. Moreover, dairy fermented products are frequently consumed fresh, containing therefore a complex, live microbial consortium mostly represented by lactic acid bacteria (LAB), which enter the human body and reach the gastrointestinal tract, where they can transiently interact with the resident gut microflora of the host. Several dairy and meat products typical of Mediterranean countries are obtained by traditional manufacturing procedures that often employ raw material, relying on the natural microflora preexisting in such ingredients, whose species composition reflects local environments [1]. The food microbiota of traditional fermented products is therefore extremely complex, partly specific for each food, and in constant interaction with the environmental bacterial community, including the gut microbiota of animals and humans. The most relevant LAB in fermented foods belong to the genera* Lactococcus*,* Lactobacillus, Streptococcus*,* Pediococcus*, and* Leuconostoc.* Several LAB species are also highly represented within the resident gut microbiota of healthy humans.* Lactobacillus* species, in particular, are abundant in both food and gut microbiota [2]. The interplay between these two microbial communities can greatly contribute to human health, as several foodborne species also display probiotic properties [3]. A growing body of literature suggests a link between gut microbiota composition and specific metabolic disorders, including obesity [4, 5]. Alterations of intestinal microbial composition were identified in obese human subjects as well as in animal obesity models and shown to affect host metabolism and energy storage. There are different ways by which the gut microbiota can affect host energy metabolism: one is represented by an increased level of energy extraction from the diet, involving specific bacterial phyla and classes, while others involve a direct influence on host pathways, by altering the expression of host genes, especially those involved in fat metabolism [6]. Given the capability of foodborne bacteria to transiently colonize the intestine, thus influencing resident gut microflora composition, it would be extremely important to identify the mechanisms underlying the effects of a complex foodborne microbial consortium on host energy metabolism, which are still poorly understood. One of the main obstacles towards this goal is the lack of simple model organisms suitable for these purposes. In this work we tested the nematode* Caenorhabditis elegans* as a simple animal model to evaluate the effects of a complex food-derived microbiota on well characterized metabolic pathways. * C. elegans* is of great value in several fields of biological research since many of its pathways are conserved in humans. It is widely used as a model organism for research on aging, development, and neurodegenerative diseases. In particular, for aging studies, the nematodes have the advantage of a short and reproducible lifespan and ease of cultivation.* C. elegans* is a differentiated multicellular organism with a nervous system, reproductive organs, and digestive apparatus. Furthermore, it has a simple structure and a short life cycle (less than 3 days) and can be infected by different human pathogens that can replace the regular* Escherichia coli* food source. Dietary sources, such as bacteria, play an important role in the control of* C. elegans* lifespan [7], and nematodes exhibit a decreased lifespan when subjected to a diet of pathogens compared to those fed with nonpathogenic laboratory microbes, such as auxotrophic strains of* E. coli*. Increasing evidences have revealed that bacterial metabolism has a great impact on host pathways, for example, in the case of folate or nitric oxide metabolism [8, 9]. The nematode* C. elegans* has been used in several studies to identify and characterize evolutionarily preserved traits associated with host-pathogen interactions [10] and also supplies the possibility for fast large-scale and economically feasible* in vivo* screens of new antimicrobials, along with their mode(s) of action [11]. In recent years, the nematode has also been employed as a useful model host for a wide variety of microbes relevant for human health, including LAB and probiotics. The majority of works demonstrate that several LAB species, mostly belonging to* Lactobacillus* genus, increase the nematode lifespan, although such effects are highly strain-dependent (reviewed in [8]). On the other hand, some LAB species have also been demonstrated to exert detrimental effects on development and growth of worms [12]. It was reported that different* Lactobacillus* species, including* L. brevis* and* L. plantarum*, isolated from fermented vegetable foods, may contribute to host defenses and prolong* C. elegans* lifespan [13]. A new functional screening method was recently developed to identify and characterize new potential antioxidant probiotic bacteria, revealing that a strain of* L. rhamnosus* could protect the nematode against oxidative stress [14]. In a recent work, authors provide evidence that a strain of* L. salivarius*, isolated from faeces of centenarian subjects, induced longevity in nematodes by dietary restriction [15]. Moreover, a study investigating the effect of* Bifidobacterium infantis* on* C. elegans* longevity found a modest dose-dependent lifespan extension when* B. infantis* was added to* E. coli* conventional food source, and such effects were also observed when nematodes were fed on cell wall or protoplast fractions of* B. infantis*, suggesting the involvement of host protective pathways activated by bacterial cell wall components, rather than the production of bacterial metabolites or gut colonization [16]. However, the use of* C. elegans* as a model organism for the study of probiotic or foodborne bacteria has been restricted to the analysis of single bacterial strains, often deriving from collections, while few data are available on natural, uncharacterized strains, as well as on complex foodborne microbiota as a whole. Mozzarella di Bufala Campana (MBC) is an example of traditional Italian PDO (Protected Designation of Origin) cheese that is consumed fresh within 2 weeks from production and contains high titer of live and complex microflora [17]. In this work we provide evidence that feeding* C. elegans* with a LAB consortium derived from MBC influences longevity, larval development, fertility, lipid accumulation, and gene expression related to obesity in this model organism, as supported by transcriptional analysis of some genes involved in fat metabolism.

## 2. Materials and Methods

### 2.1. Cheese Microbiota Preparation, Bacterial Strains, and Growth Conditions

10 g of Mozzarella di Bufala Campana (MBC) samples were diluted in 90 mL sodium citrate solution (2% w/v) and homogenized in a BagMixer400 (Interscience, France). 60 *μ*L of homogenate was inoculated in 50 mL of MRS medium (Oxoid Ltd., England) and incubated at 37°C for 48 h under anaerobic conditions (Anaerocult A, Merck, Germany), to obtain a bacterial titer of about 1 × 10^10^ Cfu/mL, corresponding to OD_600_ = 3. Bacterial counts were obtained by serial dilution in quarter-strength Ringer's solution, followed by plating on MRS agar. Plates were incubated at 37°C for 48 h under anaerobic conditions. Independent colonies displaying different morphologies were isolated from the plates, grown as described above, and stored at −80°C in 15% (v/v) glycerol.* Lactobacillus rhamnosus* GG (LGG, ATCC53103) was used as probiotic control where indicated.

### 2.2. *C. elegans* Strains and Growth Conditions

Worms were cultured as described previously [18] and grown at 16°C supplemented with the* Escherichia coli* OP50 (originally obtained from the Caenorhabditis Genetics Center), unless otherwise indicated. The* C. elegans* strains used in this study are* daf-16* (mu86) mutant and Bristol N2 as standard wild type strain, provided by the Caenorhabditis Genetic Center.

Worms were fed with* E. coli* OP50 or LGG or with foodborne microbiota. LAB consortium and LGG cultures were daily prepared as follows: aliquots of frozen cheese homogenate or frozen LGG stock were inoculated in MRS medium and grown at 37°C overnight under anaerobic conditions. Similarly, an aliquot of OP50 frozen stock was inoculated in LB medium and grown at 37°C overnight under aerobic conditions. Afterwards, each type of bacterial lawn was prepared by spreading 25 *μ*L of the bacterial suspension in M9 buffer, corresponding to 10 mg of bacterial cells, on nematode growth medium (NGM) modified to be peptone-free (mNGM) in 3.5 cm diameter plates, according to Ikeda et al. (2007) [19].

### 2.3. Lifespan Assays

Synchronized N2 adults were allowed to lay embryos for 2 h directly on mNGM, covered with the indicated bacterial lawns, and then sacrificed. All lifespan assays started when the progeny became fertile (t0). Animals were transferred to new plates spread with fresh lawns and monitored daily. They were scored as dead when they no longer responded to gentle prodding with a platinum wire. Worms that crawled off the plates were not included in the analysis.

### 2.4. Pharyngeal Pumping Assay

Pharyngeal pumping was analyzed as described by Uccelletti et al. [22] under Zeiss Axiovert 25 microscope by counting the number of contractions (defined as backward grinder movements in the terminal bulb) on 40 animals for each treatment, during five periods of 30 s. The analysis was performed on L4 wild-type worms, grown on LAB or* E. coli* (control) starting from embryo stage. The experiment was performed after 6 days from egg hatching.

### 2.5. Brood Size Measurement

Progeny production was evaluated according to Zanni et al. [23] with some modifications. Briefly, synchronized worms obtained as above were grown on mNGM plates seeded with bacteria and then were allowed to lay embryos at 16°C. Next, animals were transferred onto a fresh bacteria plate every day, and the number of progeny was counted with a Zeiss Axiovert 25 microscope. The procedure was repeated for 4 days until the mother worms stopped laying eggs. Each day the progeny production was recorded and was compared with the OP50- or LGG-fed nematodes.

### 2.6. Body Size and Embryos Length Measurement

Embryos and individual animals were photographed after 3, 4, and 5 days from egg hatching using a Leica MZ10F stereomicroscope connected to Jenoptik CCD camera. Length of embryos or worm body was determined by using the Delta Sistemi IAS software and compared to OP50- or LGG-fed worms. At least 30 nematodes or embryos were imaged on at least three independent experiments.

### 2.7. Lipid Droplets Visualization

Approximately 100 L4 nematodes, grown on LAB, LGG, or OP50 containing mNGM plates, were suspended in 1 mL of M9 buffer and washed three times. Subsequently, worms were incubated with a solution of 6.7 *μ*g/mL BODIPY 493/503 (Life technologies) for 20 min, as indicated in the vital staining protocol reported in [24]. Afterwards, worms were mounted onto 3% agarose pads containing 20 mM sodium azide and observed with a Zeiss Axiovert 25 microscope. BODIPY images were acquired using identical settings and exposure times to allow direct comparisons.

### 2.8. Real-Time qPCR

Total RNA from LGG- or LAB-fed L4 worms was isolated with Trizol reagent (Invitrogen) and then digested with 2 U/*μ*L DNAse I (Ambion). 800 ng of each sample was reverse-transcribed using oligo-dT and enhanced Avian reverse transcriptase (SIGMA, Cat. number A4464), according to manufacturer's instructions. For real-time qPCR assay, each well contained 2 *μ*L of cDNA used as template, SensiMix SYBR & Fluorescein Kit purchased from Bioline, and the selective primers (200 nM) designed with Primer3 software and reported in Table 1. All samples were run in triplicate. I Cycler IQ Multicolor Real-Time Detection System (Biorad) was used for the analysis. The real-time qPCR conditions are described by Gorietti et al. [25]. Quantification was performed using a comparative CT method (CT = threshold cycle value). Briefly, the differences between the mean CT value of each sample and the CT value of the housekeeping gene (*ama-1*) were calculated: ΔCT_sample_ = CT_sample_ − CT_*ama*-1_. Final result was determined as 2^−ΔΔCT^ where ΔΔCT = ΔCT_sample_ − ΔCT_control_.

### 2.9. Estimation of Bacterial CFU within the Nematode Gut

For each experiment, 10 animals at L4 stage and at 8 days of adulthood were washed and lysed according to Uccelletti et al. [11]. Whole worm lysates were plated onto MRS-agar plates. The number of CFU was counted after 48 h of incubation at 37°C, anaerobically.

### 2.10. Bacterial Species Identification and Strain Typing

Total bacterial DNA was obtained from colonies or inoculum by microLYSIS (Microzone, Canada), according to the manufacturers' instructions, and used as template for PCR amplifications. Strain typing was performed by rep-PCR fingerprinting with the GTG_5_ primer, as described by Gevers et al. [21] (Table 1). The eubacterial P0-P6 primer pair (Table 1, Invitrogen Life Technologies, Italy) was used to amplify 16S rRNA gene fragments [20]. PCR mixtures contained 200 *μ*M/each dNTPs, 1 *μ*M/each forward and reverse primer, 2 mM MgCl_2_, and 2 U Taq DNA polymerase (Invitrogen, Italy) in the supplied buffer. The PCR products were eluted from gels and purified by NucleoSpin Extract II Purification Kit (Macherey-Nagel, Italy) and sequenced with the forward primer (Eurofins MWG Operon Sequencing Service, Germany). Taxonomic identification was performed by comparing the DNA sequences of amplified 16S rDNA fragments with those reported in the Basic BLAST database [26]. Representative isolates belonging to each rep group were subjected to 16S rDNA amplification and sequencing in order to identify the species.

### 2.11. Statistical Analysis

Experiments were performed at least in triplicate. Data are presented as mean ± SD, and Student's *t*-test or one-way ANOVA analysis coupled with a Bonferroni post test (GraphPad Prism 4.0 software) was used to determine the statistical significance between experimental groups. Statistical significance was defined as ^∗^ *P* < 0.05, ^∗∗^ *P* < 0.01, and ^∗∗∗^ *P* < 0.001.

### 2.12. GenBank Accession Numbers

Nucleotide sequences of amplified 16S rDNA from representative isolates were submitted to GenBank, and the corresponding accession numbers are reported below:

 t0-1: KM272561
 t0-11: KM272562
 t0-12: KM272563
 t0-15: KM272564
 t0-3: KM272565
 t0-36: KM272566
 t0-51: KM272567
 t0-6: KM272568
 L4-1: KM272569
 L4-16: KM272570
 L4-22: KM272571
 L4-23: KM272572
 L4-39: KM272573
 L4-4: KM272574
 L4-6: KM272575
 L4-9: KM272576.

## 3. Results

### 3.1. Effect of LAB Microbiota Supplementation on Nematode Lifespan and Development

**Figure 1:**
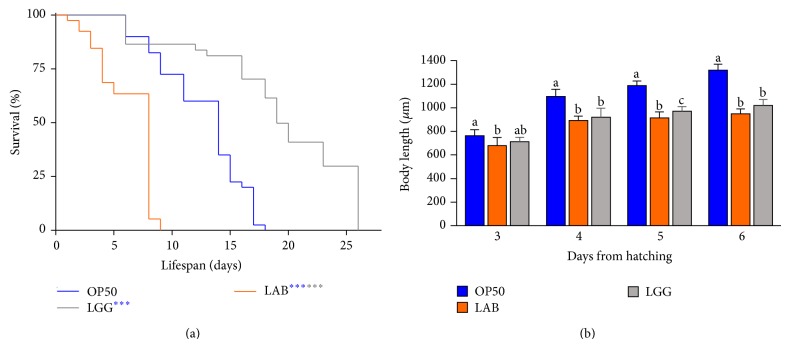
*Effect of MBC microbiota on nematode lifespan and adult body size.* (a) Kaplan-Meier survival plot of N2 worms fed with LAB consortium. Lifespans of* E. coli* OP50- and LGG-fed animals are reported as controls; *n* = 60 for each data point of single experiments. (b) Effect of LAB feeding on* C. elegans* body size. Worms were grown in the presence of OP50, LGG (controls), or MBC-derived LAB and their length was measured from head to tail at the indicated time points. The blue or grey asterisks indicate the *P* values (log-rank test) against OP50 or LGG, respectively.

Our goal was to study the effects of a dietary source consisting of a foodborne microbial consortium, represented by the LAB component of MBC cheese microbiota, on* C. elegans* physiology. Lifespan analysis was performed by monitoring LAB-fed N2 wild type animals, starting from the L1 larval stage, in comparison with control worms grown on* E. coli* OP50 or on a claimed commercial probiotic strain, namely,* L. rhamnosus* GG (LGG). Intriguingly, the MBC microbiota diet induced a relevant reduction in* C. elegans* longevity in contrast to an increased lifespan observed in the LGG-fed worms (Figure 1(a)). Such effects, expressed as the time at which 50% of the worm population is dead, were recorded at days 8 and 19 in LAB- and LGG-fed nematodes, respectively, in comparison with day 14 in* E. coli* fed animals. Notably, lifespan shortening appeared to be dependent on the viability of the microbiota used as food source. Indeed, dietary administration of heat killed LAB did not produce any effect on nematode lifetime (data not shown). Microscopic observations of LAB-fed animals resulted in a size reduction with respect to OP50-fed animals along all the developmental stages, reaching in length 70% of the control during the adulthood (Figure 1(b)). To determine whether longevity and size reduction could originate from inefficient food uptake, a pumping rate analysis was performed. However, no difference was recorded between nematodes grown on LAB lawns and the OP50-fed worms (data not shown).

### 3.2. LAB Microbiota Diet Influences Fertility and Host Energy Metabolism

**Figure 2:**
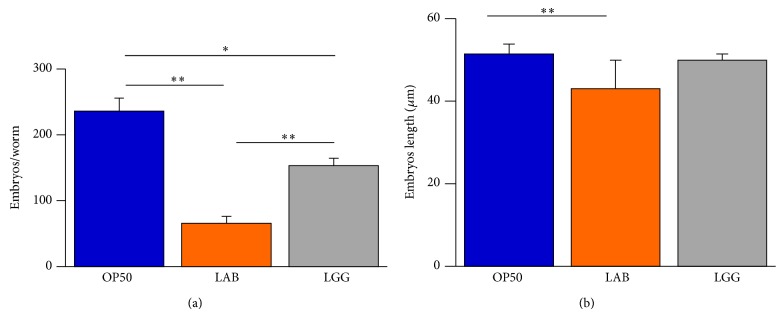
*Effect of MBC microbiota on nematode fertility.* (a) Average embryos production per worm of OP50-, LGG-, or LAB-fed animals. Bars represent the mean of three independent experiments. (b) Measurements of embryos length derived from worms fed with the indicated bacteria from the L1 stage.

Our attention was then focused on exploring possible F1 alterations induced in* C. elegans* by MBC microbiota as dietary source. To this aim, progeny production was evaluated. The brood size of LAB-fed worms was strikingly decreased, with a 72% reduction of progeny number compared with animals grown with the* E. coli* food source. A reduction was also observed for the progeny derived from LGG-fed animals, although to a lesser extent (Figure 2(a)).

In addition, MBC microbiota administration led to a decreased size of embryos as compared to the OP50 control that was not observed in the case of LGG-fed animals (Figure 2(b)).

**Figure 3:**
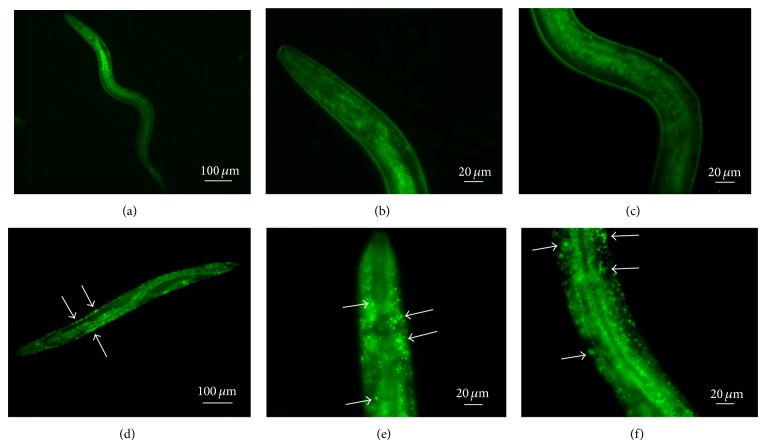
*Visualization of lipid droplets.* BODIPY staining of L4* C. elegans* animals grown in the presence of LGG (a, b, c) or LAB (d, e, f) as food sources.

Intriguingly, microscope observation after 3 days from egg hatching and during the adulthood showed a higher transparency in LAB*-*fed animals with respect to OP50-fed worms (data not shown). Since this phenotype called “Clear” has been reported in animals showing lipid homeostasis defects [27], we used the lipophilic BODIPY dye to investigate fat storage alterations [28]. LAB diet induced in the animals an enhanced fluorescence as compared to* E. coli* fed worms (data not shown), thus confirming an increase in lipid accumulation. However, LAB-fed animals (Figures 3(d), 3(e), and 3(f)) displayed large aggregates of lipid bodies with respect to LGG-fed nematodes (Figures 3(a), 3(b), and 3(c)).

**Figure 4:**
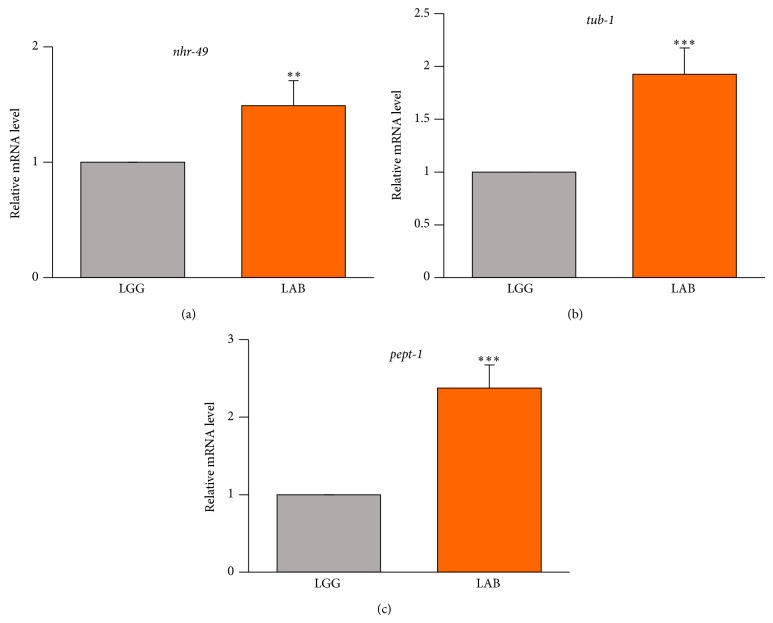
*RT-qPCR analysis of lipid metabolism genes.* Expression of (a)* nhr-49*, (b)* tub-1*, and (c)* pept-1* genes in LGG- or LAB-fed animals after 3 days from hatching. Histograms show the expression of lipid metabolism related genes detected by real-time PCR.

In order to identify the genes possibly responsible for the alterations induced by the LAB consortium with respect to LGG, real-time qPCR analysis was performed for* nhr-49*,* pept-1*, and* tub-1* genes, whose involvement in lipid metabolism and obesity-related phenotypes have been described [29–31]. The results showed that the MBC microbiota diet induced an increased transcription of all tested genes with respect to nematodes fed with the LGG diet (Figure 4).

**Figure 5:**
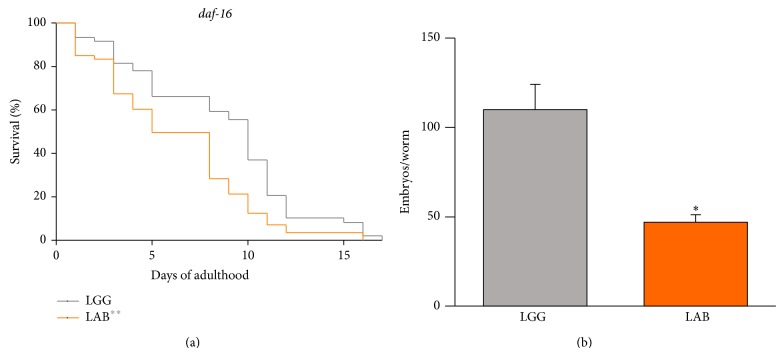
*Effect of MBC microbiota on daf-16 mutant.* (a) Kaplan-Meier survival plot of* daf-16* worms fed with LAB consortium. Lifespan of LGG-fed animals is reported as control; *n* = 60 for each data point of single experiments. (b) Average embryos production per worm of LGG- or LAB-fed* daf-16* animals. Bars represent the mean of three independent experiments. The grey asterisks indicate the *P* values (log-rank test) against LGG.

Since* daf-16* is one of the major regulators of fat metabolism [32], we sought to test whether the LAB diet could alter fat metabolism by modulating this factor. To this aim, we fed loss-of-function* daf-16* mutant nematodes with LAB and assessed the lifespan, the fertility, and the fat storage. The effect of the LAB consortium on fat deposition (data not shown) and on the lifespan of the mutant animals was similar to that observed with the N2 strain (Figure 5(a)). Moreover, LAB supplementation induced a progeny reduction analogous to the wild type animals (Figure 5(b)).

### 3.3. Colonization Capacity of Microbiota and Species Characterization

**Figure 6:**
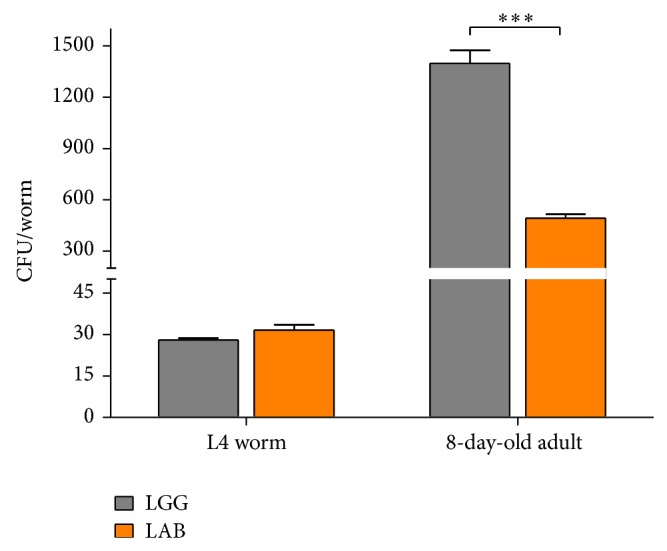
*Bacterial colonization capacity.* Bacterial colony forming units (CFU) recovered from nematodes were obtained by plating whole lysates of L4 and 8-day-old adults fed LGG (grey bars) or LAB (orange bars). Bars represent the mean of three independent experiments.

Intestinal colonization experiments were performed to determine whether the microbiota was consumed by the worms. To this aim, worms fed LAB or LGG were lysed at different time points and the resulting whole lysates, plated on MRS and grown as described (see Section 2), were used to evaluate the bacterial colony forming units (CFU). Results, reported in Figure 6, demonstrated that both diets increased the intestinal CFUs along the lifespan. However, the CFU number relative to MBC microbiota diet resulted to be about 3-fold less than that relative to LGG diet at 8 days of adulthood (Figure 6).

In order to identify the predominant bacterial species most likely involved in inducing the observed phenotypes in* C. elegans*, the MBC microbiota was subjected to species identification. We first examined the isolates associated with different colony morphology (i.e., large and smooth, large and rough, and medium), deriving from plates obtained by serial dilution of the homogenate inoculum used to feed the nematodes (see Section 2). Microscope observation of 10 isolates representative of each colony morphology revealed the presence of long, filamentous, nonmotile rods (large and smooth colonies), short nonmotile rods (large and rough colonies), and ovoid forming cocci (medium colonies) (data not shown). 16S rDNA amplification and sequencing identified them as* Lactobacillus delbrueckii*,* L. fermentum*, and* Leuconostoc lactis*, respectively.

**Figure 7:**
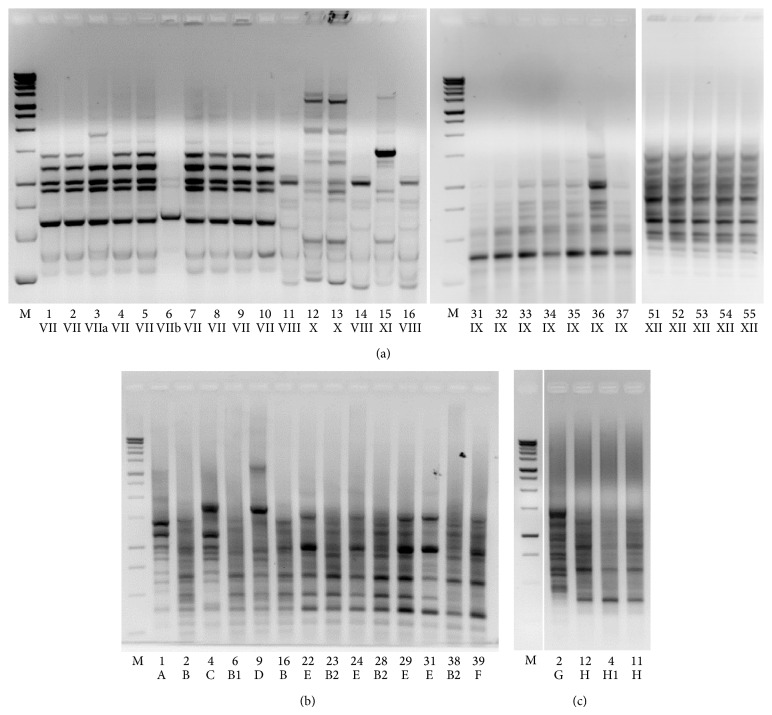
*Biodiversity of nematode intestinal microbiota.* Rep fingerprinting profiles of bacterial colonies randomly chosen after plating MBC homogenates used to feed worms (a) or nematode intestinal lysates at L4 stage (b) and 8 days of adulthood (c) following LAB microbiota supplementation. Arabic numerals indicate each isolate, while roman numerals or letters identify rep groups. M: 1 kb DNA ladder, Promega. Only representative rep groups are shown in panels a and c.

A more in-depth analysis concerned strain typing of a representative number of isolates, ranging from 20 to 40 colonies, performed at time point 0 (representing the initial microbial LAB consortium used to feed the worms), as well as at different nematode stages (representing the intestinal microbial community of* C. elegans* fed MBC microbiota). In this latter case, worms supplemented until L4 stage or 8 days of adulthood with cheese microbiota were washed and lysed, and the resulting lysates were plated on MRS in order to isolate bacterial colonies. Strain typing was carried out by rep fingerprinting (see Section 2) and the results are shown in Figure 7. Isolates displaying the same fingerprinting profile were assigned to a single rep group. Rep-PCR amplification identified 8 distinct rep groups, out of 28 isolates analyzed, at time point 0 h (Figure 7(a)), while at L4 stage (Figure 7(b)) and at 8 d (Figure 7(c)) 8 rep groups, out of 40 isolates analyzed, and 3 rep groups, out of 34, were identified, respectively. Only representative rep groups are shown in Figures 7(b) and 7(c). Species assignments were defined on the basis of sequencing results (see Section 2) or restriction digestion (data not shown) of 16S rDNA amplified from representative isolates. Correlations between the species and rep groups, as well as their distribution in the worm gut at different stages or at time point 0, are summarized in Table 2. Overall, we observed an equal distribution of* L. delbrueckii*,* L. fermentum*, and* Leuc. lactis* in the initial microbial consortium, while a progressive decreasing of* Leuc. lactis* was observed in the worm gut microbial community at the observed time points. In particular, only* L. fermentum* and* L. delbrueckii* were recovered from nematode lysates at 8 days of adulthood, with a prevalence of the* L. delbrueckii* species (28 isolates out of 34) (Table 2).

## 4. Discussion

In the present work, we have evaluated the impact on* C. elegans* physiology of a complex, foodborne microbial consortium derived from a traditional fermented cheese. To this aim, nematodes were fed MBC microbiota from hatching throughout development. Longevity analysis demonstrated that LAB-fed animals exhibited a reduced lifespan with respect to control worms, fed conventional* E. coli* OP50 or the LGG probiotic strain. Such effect was not attributable to inefficient food uptake and was dependent on LAB viability. An infection-like process exerted by the consortium could be hypothesized; however, nematodes grown with MBC microbiota did not show intestinal lumen distension (data not shown), a characteristic sign of pathogenesis [33]. Moreover, lifespan analysis in the immunocompromised* daf-16* mutant revealed that LAB diet induced a reduction in the lifetime to the same extent of that observed for* C. elegans* wild type strain, reinforcing the idea that no infection process took place in LAB-fed worms.

The observed effect on longevity was quite surprising, since in the majority of previously published articles, LAB have been shown to increase the nematode lifespan [13–16, 19]. However, a negative effect exerted by* L. salivarius*,* L. reuteri*, and* Pediococcus acidilactici* on the development and growth of the worm was also recently reported [12]. Such conflicting results could be explained by assuming that distinct species and strains can promote differential effects on* C. elegans*. Moreover, it is important to consider that in some cases the experiments have been performed with heat killed bacteria [13, 15] and that in most published studies LAB were supplied to adult worms, rather than the embryos.

We consider a key aspect that bacteria must be viable to exert these effects, since fermented dairy products, which are usually consumed fresh, are characterized by high titers of live microflora, potentially capable of colonizing the human gut. In addition, to evaluate the effects of foodborne microbes it would be “more physiological” to study the whole complex microbiota* per se* rather than the single species that could mask the effects of the consortium. Complex bacterial environments can be composed indeed of several underrepresented species, difficult to isolate.

Analysis of MBC consortium in terms of species composition resulted in the identification of three predominant species,* L. fermentum*,* L. delbrueckii*, and* Leuc. lactis*, in agreement with previous findings [17] which have never been tested in* C. elegans* so far. The same species were also found associated with worm gut after ingestion, with different distributions relative to the time points analyzed. Taken together, these results suggest that the overall initial cheese microbiota composition is maintained in the nematode intestine during the development while, at 8 days of adulthood, only* L. delbrueckii* and* L. fermentum* are still capable of surviving in the worm gut. In particular, prevalence of* L. delbrueckii* was observed. On the other hand,* Leuc. lactis* appears to be no longer able to colonize worm intestine. Several factors contribute to bacterial competition for colonization in* C. elegans* gut, such as selection by the host and strain-specific characteristics [34].* C. elegans* gut colonization is still an open question, and several works report a correlation between bacterial colonization and nematode lifespan, as well as defence response [35, 36].

Previous findings suggest that both* Enterococcus faecalis* and* E. faecium* accumulate to high titers in the nematode gut lumen during infection process, although only* E. faecalis* was able to kill adult worms [37]. Conflicting results have been reported by Forrester et al., who demonstrated that the foodborne pathogen* Listeria monocytogenes*, although exerting deleterious effects on* C. elegans*, did not persist in the nematode intestine [38], suggesting that a complex set of mechanisms regulate host-bacteria interactions. Similarly, a reduced colonization capacity has been observed in the case of MBC microbiota with respect to LGG. Further studies are therefore necessary to unravel such mechanisms.

Beyond the effects on lifespan, we observed that LAB- and LGG-fed worms displayed fat accumulation, as revealed by BODIPY staining. In* C. elegans*, fat is mainly stored in intestinal and hypodermal lipid droplets [39, 40]. In particular, LAB-fed nematodes displayed larger lipid bodies with respect to LGG-fed worms, suggesting that foodborne microbial consortium can have a higher impact on host fat metabolism.

Foodborne microbes are introduced in the human body through the food chain, and those capable of surviving the harsh conditions of the upper GI tract can reach the colon, where they can interact and modify the autochthonous gut microbiota. However, the contribution of foodborne bacteria to the modulation of host energy metabolism is still unclear. One of the mechanisms by which the gut microbes can lead to metabolic disorders is the alteration of expression of host genes involved in energy expenditure and conservation [41]. We found that genes previously described as responsible for obesity phenotypes in* C. elegans*, that is,* pept-1*,* nhr-49*, and* tub-1*, are induced in MBC microbiota-fed worms, demonstrating for the first time the capability of foodborne microbes to alter lipid metabolism and accumulation.

It has been reported that different* E. coli* strains significantly affect fat storage levels through the involvement of the intestinal peptide transporter, Pept-1 [42]. The fact that LAB-fed worms showed higher level of* pept-1* transcript with respect to LGG-fed animals suggests that the uptake of di- and tripeptides derived from foodborne microbiota could be not enough to guarantee the building blocks for protein synthesis. However, we can not exclude that reduced functionality of the transporter could be caused by an altered intestinal cell pH, originating from LAB metabolism inside the worm gut that leads to an increased mRNA level as a compensatory mechanism. This hypothesis is in agreement with the fact that* pept-1* mutants showed phenotypes similar to those observed in LAB-fed nematodes [43].

Foodborne microbiota induced in* C. elegans* also increased transcription of the nuclear hormone receptor gene,* nhr-49*, although to a lesser extent compared to the other genes tested, namely,* pept-1* and* tub-1*. This gene encodes a transcription factor localized within nematode body wall muscle cells [44] and involved in the control of pathways that regulate fat consumption and maintain normal balance of fatty acids saturation, by modulating the expression of genes involved in fatty acid beta-oxidation [29]. It could be speculated that the increased* nhr-49* transcript levels in LAB-fed animals represent an attempt to decrease the fat storages by increasing the beta-oxidation process.

Lipid accumulation has been connected in* C. elegans* with the sensory cilia, implicated in sensing the chemical and/or physical extracellular environments. Tub-1, the transcription factor homologous to the mouse protein Tubby, results to be localized to the axons, dendrites, and cilia [45] and influences fat metabolism [46]. Increased levels of* tub-1* transcript were also found in LAB-fed nematodes, suggesting a link between the sensory neuron and the diet that will deserve further investigations.

Taken together, these results suggest that supplementation of nematodes with a foodborne microbiota influences host energy metabolism. Both microbiota- and LGG-fed animals showed reduced progeny; this can be in agreement with the fact that worms spend part of their energy on reproduction. Embryos use yolk transferred from the intestine to the gonadal arms as a primary food source [47]; we therefore can hypothesize that LAB and LGG diet may alter the reproductive behaviour indirectly through modifications of fat stores.

Intriguingly, the predominant species identified in LAB consortium used to feed the nematodes and triggering obesity-like phenotypes belong to Firmicutes phylum. Both diet- and genetic-induced obesity are associated with an imbalance between Bacteroidetes and Firmicutes, the two major phyla of gut bacteria, with Firmicutes prevailing in obese subjects [48].

Studies aimed at investigating the role of gut bacteria in metabolic disease progression have greatly profited from gnotobiotic animal models [6]. However, the availability of simpler model organisms would be extremely valuable in terms of ease of cultivation, reduced costs, and ethic issues. We have provided evidence that foodborne microbes can colonize* C. elegans* gut and modulate host gene expression. Overall, our results could represent a first step towards the identification of metabolic pathways influenced by foodborne bacteria.

## 5. Conclusions

*C. elegans* has been extensively used in biology research and, most recently, some studies have taken advantage of this model to study host-microbiota interactions using isolated foodborne bacteria or probiotic strains.

Herein we found that supplementation of nematodes with a foodborne microbiota, derived from a traditional fermented dairy product, influences longevity and fertility as well as lipid accumulation and gene expression related to obesity, as supported by transcriptional analysis of some genes involved in fat metabolism. We provide evidence that, in foodborne microbiota-fed worms, expression of genes encoding transcriptional factors involved in fatty acid beta-oxidation or nutrient sensing and influencing fat metabolism and storage is induced, demonstrating the capability of foodborne microbes to alter host lipid metabolism and accumulation. Moreover, we found that the main bacterial species (*L. fermentum*,* L. delbrueckii*, and* Leuc. lactis*) characterizing the consortium are able to colonize worm gut.

Overall our work extends the applicability of such model in the field of host-microbiota interaction by evaluating the influence exerted by a cheese derived LAB consortium on different physiological aspects of the nematodes.

## Figures and Tables

**Table 1:** Primers used in standard and RT-qPCR assays.

Primer name	Primer sequence (5′-3′)	Target gene	Organism	PCR assay	Reference
P0 P6	GAGAGTTTGATCCTGGCT CTACGGCTACCTTGTTAC	16S-rDNA	Bacteria	Standard	[20]
GTG_5_	GTGGTGGTGGTGGTG	Genomic repetitive elements	Bacteria	Standard	[21]
pept-1 For pept-1 Rev	GTGTTCGGAGAAGTATCTCG CAAGAGCACAGTCGTGAGTA	*pept-1* GenBank accession number NM_076686	*C. elegans *	Real-time	This work
tub-1 For tub-1 Rev	CCACAGCAAGTTCAAGAGTC AGCCACTACATCAGTGTTCC	*tub-1* GenBank accession number NM_063309	*C. elegans *	Real-time	This work
nhr-49 For nhr-49 Rev	GCTCTCAAGGCTCTGACTC GAGAGCAGAGAATCCACCT	*nhr-49* GenBank accession number NM_001264305	*C. elegans *	Real-time	This work
act-1 For act-1 Rev	GAGCGTGGTTACTCTTTCA CAGAGCTTCTCCTTGATGTC	*act-1* GenBank accession number NM_073418	*C. elegans *	Real-time	This work

**Table 2:** Bacterial species and related rep groups associated with the nematode gut at different time points after the initial supplementation. Time point 0 refers to the microbial community used to feed the worms.

Time point	Species	Rep group	*N* isolates	Total isolates
0 h	*L. delbrueckii* *L. fermentum* *Leuc. lactis *	VIII, IX X, XI, XII VII, VIIa, VIIb	9 8 10	28

L4	*L. delbrueckii* *L. fermentum * *Leuc. lactis *	B2, E, F B, B1, D A, C	20 14 6	40

8 d adults	*L. delbrueckii* *L. fermentum *	H, H1 G	28 7	34
